# Molecular epidemiology of bovine leukemia virus in cattle and phylogenetic analysis for determining its prevailing genotype in Khyber Pukhtunkhwa, Pakistan

**DOI:** 10.1080/10495398.2025.2486029

**Published:** 2025-04-17

**Authors:** Farida Tahir, Umer Sadique, Farkhanda Tahir, Mikhlid H. Almutairi, Abdulwahed Fahad Alrefaei, Shabana Naz, Rifat Ullah Khan, Naseer Khan Momand, Marco Ragni

**Affiliations:** a College of Veterinary Sciences, Faculty of Animal Husbandry and Veterinary Sciences, The University of Agriculture, Peshawar, Pakistan; b Department of Biochemistry, Abdul Wali Khan University, Mardan, Pakistan; c Department of Zoology, College of Science, King Saud University, Riyadh, Saudi Arabia; d Department of Zoology, Government College Univeristy, Faisalabad, Pakistan; e Animal Science, Nangarhar Univeristy, Kabul, Afghanistan; f Soil, Plant and Food, University of Bari, Aldomoro, Itay

**Keywords:** Bovine leukemia virus, cattle, enzootic bovine leukemia, genotypes, nested PCR, phylogenetic analysis

## Abstract

This research focused on assessing the molecular prevalence of Bovine Leukemia Virus (BLV) in different cattle farms throughout Khyber Pakhtunkhwa and characterizing the dominant BLV genotypes by analyzing partial sequences of the gp51 gene. A total of 1,250 blood samples were collected from cattle of both sexes, various age groups (<1 year, 1–3 years, 3–5 years, and >5 years), and different breeds (Friesian, Jersey, Sahiwal, Achai, and crossbred) from multiple cattle farms. Of the 1,250 samples tested, BLV was detected in 136 (10.88%) using nested PCR. Risk factor analysis revealed a significantly higher prevalence of BLV in exotic breeds and older cattle. To confirm the findings and genotype the BLV isolates, four PCR-positive samples were sequenced. Phylogenetic analysis identified the isolates as belonging to genotype I, closely related to GI BLV isolates from Japan. Furthermore, the isolates in this study formed a tightly clustered group, suggesting a common origin from an earlier virus introduced into the host population in the study area.

## Introduction

Enzootic bovine leukosis (EBL), commonly known as bovine leukosis, is caused by bovine leukemia virus (BLV), a member of the genus *Deltaretrovirus* within the family *Retroviridae.*^1–3^ Cattle and water buffalo are considered natural hosts for BLV infection.^4^ Though some animal species (e.g., deer, llamas, capybaras, rhesus monkeys, cat, chkcens, chimpanzees, and antelopes) are susceptible to BLV, natural infections in wild populations have not been conclusively identified.^4–6^ The virus has also been detected in sheep (*Ovis aries*).^7^^,^^8^ Under experimental conditions, BLV infection has been observed in goats, pigs, chickens, rats, and rabbits, where it exhibits a shorter latency period, increased virulence, and higher transmission rates compared to cattle.^9^ The estimated inheritability of bovine leukosis in populations of Holstein and Jersey cattle is around 0.08%, suggesting that genetic vulnerability contributes to the incidence of BLV in some breeds of cattle.^10^ BLV infection is present throughout the world and prevalence ranges from as low as less than 20% in Asian and Middle Eastern countries to as high as 80% in North and South America. The endemic form of BLV negatively affects the economy in the form of trade restrictions, production losses, culling of the diseased animals, and affecting the carcass quality during slaughtering. Furthermore, the afflicted animals have weakened immune systems and are more vulnerable to various diseases.^11^

BLV is a single-stranded, diploid RNA virus with a genome consisting of 8714 nucleotides.^1^ Several studies have shown that the **env** gene undergoes genetic variation, which can be valuable for phylogenetic analysis and the classification of BLV isolates.^12^ Env gene’s nucleotide sequence and amino acid composition serve as helpful genomic markers of BLV for research on the disease’s dissemination and to identify the existence of various genotypes that are associated with regional origin.^13^ As a result, sequencing and phylogenetic analysis of partial and full-length gp51 env genes have led to the identification of at least 12 BLV genotypes. Genotype 1 is the most frequently found in the US, Japan, and Korea along with genotype 3.^14^ South America has been home to genotypes 1, 2, 3, 4, 5, 6, and 9 (genotype 9 particularly in Bolivia). In Eastern Europe and Russia, genotypes 4, 7, and 8 are prevalent whereas in China, Vietnam, Thailand, and Myanmar genotype 10 is prevalent.^15^ G6 genotype has been discovered in South American countries such as Brazil, Argentina, Paraguay, Peru, and Bolivia,^16^ as well as Asian nations like the Philippines, Thailand, and India^17^^,^^18^ whereas Genotype 11 have been documented in China.^19^ Isolates representing geographically distinct env sequences shared a high degree of sequence homology, several of them revealed the occurrence of minor point mutations.^20^ It was demonstrated that some of these mutations are linked to the pathophysiology, replication, and infectiousness of BLV.^21^^,^^22^

Effective diagnostic methods should be developed for routine and simple use to identify the infected animals to improve the detection of BLV infection and control its spread. The WOAH refers to ELISA and AGID among serological tests as the reference methods for determining BLV infection by detection of antibodies against the gp51 and p24 proteins of the BLV. ELISAs have been utilized often because of their increased sensitivity even though AGID is the gold standard.^23^^,^^24^ The development of highly sensitive and more precise molecular techniques has revolutionized the detection of BLV and other viral infections relative to serological testing.^25^ So, determining whether an animal is BLV-infected or not can be done with the help of the detection of BLV proviral DNA.^26^ Considering the constraints of various diagnostic methods for BLV detection, the present study was designed to determine the molecular epidemiology of BLV through nested PCR and partial sequencing of the gp51 gene for determining the predominant genotypes of BLV in Khyber Pakhtunkhwa.

## Materials and methods

### Sampling

A total of 1,250 blood samples were collected from government-owned farms (n = 623) and private farms (n = 627) across different districts of Khyber Pakhtunkhwa. The sampled animals included various breeds, ages, and zootechnical functions (dairy, meat, and dual-purpose). A detailed history of each animal, including clinical signs of BLV infection, was recorded using a predesigned questionnaire. The majority of animals appeared asymptomatic, but those exhibiting signs suggestive of BLV (e.g., lymphadenopathy, weight loss, or reduced milk production) were also included. Blood samples were stored at −20 °C until further processing.

### PCR

#### DNA extraction and quantification

DNA was extracted from whole blood according to the manufacturer’s recommendations (Analytikjena innuPREP DNA Micro Kit). The DNA concentration and purity were measured using the NanoDrop 1000 spectrophotometer (Thermo Fisher Scientific, Waltham, MA, U.S.A.). The extracted DNA had a concentration of 60–100 ng/µl, and the A260/280 ratio, which measures purity, was 1.6–1.7.

#### PCR and gel electrophoresis

PCR was performed to amplify the gp51 (env) gene of BLV as suggested by Fechner et al.^27^ and the World Organization for Animal Health (WOAH). A nested PCR, including internal and external pairs of primers, was used for the amplification of the target gene i.e., gp51 (env) (Table 1). For the first round of amplification, 25 μl reaction mix was prepared by mixing 5 µl PCR master mix (50 ng/μL), 10 pmol internal primers (1 µl each forward and reverse primers), 5 µl DNA template, and 13 µl PCR water. The conditions for the first round of amplification were; initial denaturation at 95 °C for 3 min, followed by 30 cycles of cycling denaturation at 95 °C for 30 sec, annealing at 58 °C for 30 sec, cyclic extension at 72 °C for 30 sec, and final extension of 72 °C for 5 min. For 2^nd^ round of amplification, internal primers (10 pmol) were used and the PCR product (1 µl) of the first round was used as a template whereas other conditions remained the same. The PCR products were analyzed on 1% agarose gel stained with ethidium bromide (3 µl) for the confirmation of the desired amplicon size (444 bp).

**Table 1.** PCR primers used in this study for bovine leukemia virus.

Organism	Target gene	Primers	Sequence (5′→3′)	Amplicon size (bp)
Bovine leukemia virus	gp51 (env)	External primes	F- TCTGTGCCAAGTCTCCCAGATA	598
			R- AACAACAACCTCTGGGAAGGG	
		Internal primers	F- CCCACAAGGGCGGCGCCGGTTT	444
			R-GCGAGGCCGGGTCCAGAGCTGG	

#### Sequencing and phylogenetic analysis

The PCR products from positive samples were sent to BGI (Hong Kong) for Sanger sequencing, following the method described by Sanger and Coulson.^28^ The acquired sequences were edited for any redundant sequences using Bioedit (version 7.2.5)^29^ before being submitted to Genbank for the accession numbers which are; OP851371-OP851374. To verify the isolates, BLAST (Basic Local Alignment Search Tool) analysis was performed, and sequences with high similarity were obtained from the National Center for Biotechnology Information (NCBI) database for evolutionary studies. For phylogenetic analysis, the DNA sequences of the BLV gene (gp51 env), which are already present in the NCBI database and have a homology of 97–100% with the accession numbers; MN167081 (Taiwan), LC512445 (Viet Nam), LC361264 (Japan), AP019595 (Japan), MG678786 (Mexico), MF817722 (Viet Nam), KY419099 (Taiwan), AB987702 (Japan), KX674371 (Saint Kitts and Nevis), LC075546 (Peru), LC164085 (Japan), KU233560 (Thailand), KP201481 (South Korea), LC005615 (Japan), LC733361 (Japan), MW926784 (Pakistan), MN928517 (Turkey), MN830811 (Costa Rica), LC498592 (Egypt), KU233528 (Thailand), MK780741 (Pakistan), KU233560 (Thailand), JN254636 (Brazil), LC498597 (Egypt), FJ808572 (Argentina) and EF065651 (Japan) were retrieved. All the sequences were aligned through Bioedit multiple alignments and a phylogenetic tree was constructed by using MEGA X.^30–32^

#### Statistical analysis

Binary logistic regression using the forward Wald statistical model (SPSS version 21) was employed to analyze data on the molecular prevalence of BLV, assessing the statistical significance of BLV prevalence and its association with various independent variables. A statistically significant value of *p* ≤ 0.05 was used to estimate the pathogen prevalence with a 95% confidence interval.

## Results

**Figure 1.**
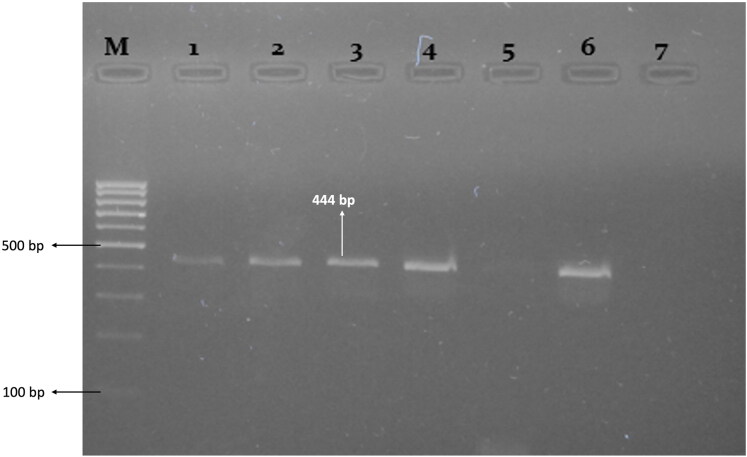
Gel picture showing 100 bp DNA marker (M), samples positive for BLV (1, 2, 3, 4, 6), having amplicon size 444 bp, and negative samples (5, 7).

The results shows that out of 1,250 samples tested, 136 (10.88%) were identified as BLV-positive. The majority of samples tested negative, indicating a relatively low prevalence of BLV in the studied population. PCR-positive samples produced amplicons of 598 bp and 444 bp when using external and internal primer pairs, respectively (Fig. 1).

### Sequencing and phylogenetic analysis

**Figure 2.**
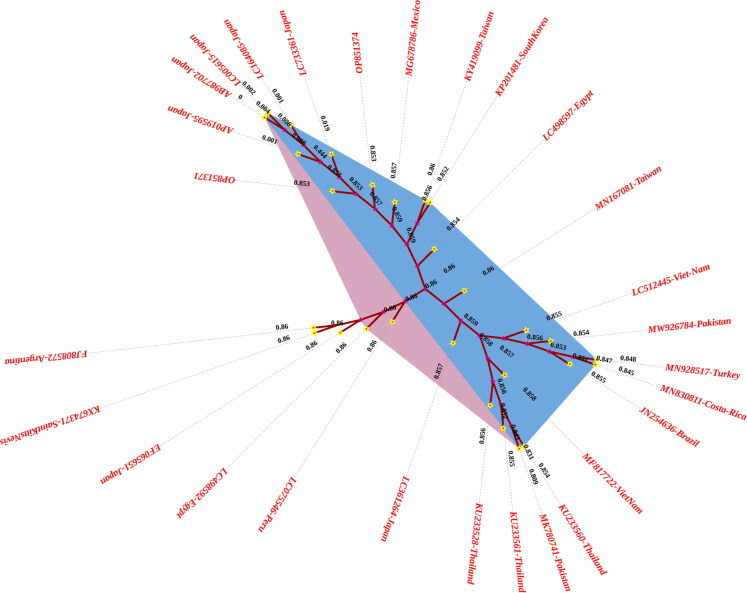
Neighbor-joining phylogenetic analysis inferred from *BLV gp51 env* sequences. Accession numbers followed by the country. Samples sequenced in the current study have accession numbers OP851371-OP851374.

The sequence analysis of the isolates in this study confirmed the amplification of a fragment of the BLV *env* gene, specifically the *gp51* region (Table 1). For phylogenetic analysis, 26 DNA sequences with high homology (97–100%) to the study isolates were retrieved from the NCBI database (Table 2). The isolates from the current study were clustered into a distinct clade, exhibiting close genetic relatedness with isolates from Japan (EF065651), and Argentina (FJ808572), whereas other sequences from South Korea, Japan, Thailand, Peru, Saint Kitts and Nevi, Taiwan, Viet Nam, Mexico, Pakistan, Turkey and Costa Rica were grouped in another clade (Fig. 2). The genotype analysis showed that the current study isolates have proximity to BLV genotype G1.

**Table 2.** Sequences retrieved from NCBI database having homology with the current study sequences, along with their accession numbers, country of origin, and genotypes.

S. No	Accession number	Country	Genotype
1	OP851371	KPK, Pakistan	
2	OP851372	KPK, Pakistan	
3	OP851373	KPK, Pakistan	
4	OP851374	KPK, Pakistan	
5	LC512445	Viet Nam	Genotype I
6	MN167081	Taiwan	Genotype I
7	LC361264	Japan	Genotype I
8	AP019595	Japan	Genotype I
9	MG678786	Mexico	Genotype I
10	MF817722	Viet Nam	G1
11	KY419099	Taiwan	G1
12	AB987702	Japan	G1
13	KX674371	Saint Kitts and Nevis	G1
14	LC075546	Peru	G1
15	LC164085	Japan	G1
16	KU233560	Thailand	G1
17	KP201481	South Korea	G1
18	LC005615	Japan	G1
19	LC733361	Japan	G1
20	MW926784	Pakistan	G1
21	MN928517	Turkey	G1
22	MN830811	Costa Rica	G1
23	LC498592	Egypt	G1
24	KU233528	Thailand	G1
25	MK780741	Pakistan	G1
26	KU233560	Thailand	G10
27	JN254636	Brazil	G1
28	LC498597	Egypt	G1
29	FJ808572	Argentina	G6b
30	EF065651	Japan	G1

### Risk factors analysis

BLV was detected in different breeds of cattle (Achai, Friesian, Jersey, Sahiwal, and Crossbred) in this study. Breed-wise molecular prevalence was higher in the Friesian breed i.e.45.1%, followed by Jersey (37.6%), Crossbred (6.3%), and Sahiwal (2.4%) whereas no positive case was detected in Achai cattle.

The relationship between cattle breed and the molecular prevalence of BLV, as determined by PCR, was analyzed using univariate regression. The results showed an odds ratio (OR) of 7.02, a confidence interval (CI) of 0.562–0.878, and a *p*-value of 0.01 (Table 3).

**Table 3.** Molecular prevalence of BLV with respect to different cattle breeds in Khyber Pakhtunkhwa.

Variables	Categories (n)	Positive (%)	OR	95%CI (lower bound-upper bound)	*p*-value
Breed	Achai (n = 329)	0	0.702	0.562–0.878	0.01
	Friesian (n = 142)	45.1			
	Jersey (n = 93)	37.6			
	Sahiwal (n = 166)	2.4			
	Crossbred (n = 520)	6.3			

Crossbred = Achai Jersey Cross, Friesian Sahiwal cross, Jersey Sahiwal cross, OR = Odd Ratio, CI = Confidence Interval.

The prevalence of BLV in male (12.47%) cattle was slightly higher than in females (10.64%) but the difference was non-significant statistically (*p* > 0.05). According to age, cattle were grouped in four different categories i.e., <1 year, 1–3 years, 3–5 years, and >5 years. Age-wise prevalence was higher in 3–5 years (12.85%), followed by >5 years (11.75%), 1–3 years (8.98%), and < 1 year (3.13%). The univariate regression analysis revealed an association between sex and age with the molecular prevalence of BLV in cattle, as determined by PCR. The results showed that sex had an odds ratio (OR) of 2.63 (CI: 1.204–5.756, *p*-value: 0.43), while age had an OR of 2.67 (CI: 2.03–3.52, *p*-value: 0.04). (Table 4).

**Table 4.** Molecular prevalence of BLV in Khyber Pakhtunkhwa with respect to sex and age of cattle.

Variables	Categories (n)	Positive (%)	OR	95%CI (lower bound-upper bound)	*p*-value
Sex	Female (n = 1118)	10.64	2.632	1.204–5.756	0.43
	Male (n = 132)	12.87			
Age	<1Year (n = 96)	3.13	2.672	2.029–3.52	0.04
	1–3Y (n = 167)	8.98			
	3–5Y (n = 179)	12.85			
	>5Y (n = 808)	11.75			

OR = Odd Ratio; CI = Confidence Interval.

## Discussion

Enzootic bovine leukosis (EBL), the most common and significant viral-induced tumor disease in cattle, is caused by the bovine leukemia virus (BLV).^1^^,^^33^^,^^34^ BLV is distinguished by its strong ability to attach to host cells, persist within the host, and integrate its DNA in proviral form into the genome, resulting in a lifelong infection. The majority of infected cattle are referred to as aleukemic (AL) and are clinically healthy, with no clinical manifestations. Approximately one-third of infected animals develop persistent lymphocytosis (PL), characterized by the polyclonal expansion of B lymphocytes, following a latency period of a few months to several years. The most apparent clinical manifestation of BLV infection is persistent lymphocytosis, which often remains stable for several years. However, in 1–5% of affected animals, it may progress to malignant lymphoma.^35^

The molecular prevalence of BLV was detected with respect to different risk factors i.e., cattle breed, sex, age, districts, farms, dam, and sire history, in the current study. The molecular prevalence was significantly higher in exotic and cross-breeds of cattle as compared to local breeds. The primary risk factor for the spread of BLV infection is the introduction of cows into herds without awareness of their infection status.^11^^,^^36^^,^^37^ The purchase of cattle from other herds in the country or other countries was not considered in this study as a risk factor, although it is clear how important it is in the spread of BLV.^38^ Dairy cow breeding in Pakistan has seen substantial changes over the past 20 years as a result of the country’s rapidly expanding demand for milk and dairy products as well as the growth of the milk processing industry. Exotic breeds are frequently preferred because of their high milk production, and cattle are imported from countries where BLV is endemic, such as USA, Australia, New Zealand, The Netherlands, and others. It’s likely that these cattle have already been exposed to BLV and may be the source of infection in the study area.^39^ Additionally, when biosecurity precautions are ignored, the unregulated exchange of adult breeding males across farms and the buying of semen straws, which is a common practice on farms, represent a danger of escalating BLV infections.^40^^,^^41^ The age-wise molecular prevalence was significantly higher in old age animals compared to young animals. A possible explanation for this difference is that older animals may have had continuous exposure for a longer period.^15^ As a herd-level disease, BLV infection can spread throughout a farm from a single infected animal and this is evident from the findings of the current study that most of the positive cases were limited to a single farm. The possible reasons for herd-wise transmission may be pooled colostrum and milk feeding, sharing milking machines among lactating animals, and close contact for a long time.^15^ Generally, herds acquire BLV mostly through horizontal transmission. The expansion of blood-sucking insect populations, which serve as the vectors for BLV transmission and assist viral dissemination, may be favored by the warm, humid subtropical climate.^42^^,^^43^ Additionally, the majority of dairy cows in the farms are housed in loose housing, which according to a prior study allows for regular animal interaction and may promote horizontal transmission within a herd.^44^ Since BLV activities have been found in cattle milk, saliva, and nasal secretions, in these conditions, iatrogenic transmission would not be prevented without the required safety precautions being taken during cattle handling, processing, and regular husbandry.^45^^,^^46^ Through artificial insemination or the in-utero method, the offspring may vertically inherit the BLV genome from a parent. Therefore, to reduce BLV transmission, adequate BLV surveillance, isolation of BLV-positive cows, and excellent management practices are necessary.

The BLV genome consists of two identical long terminal repeats, the regulatory genes *tax* and *rex*, the auxiliary genes R3 and G4, and *pro, gag, pol*, and *env* genes that code for structural proteins and enzymes.^47^ The env gene that codes for the envelop protein consists of two proteins i.e., gp51 surface glycoprotein, and gp30 transmembrane protein. Since the env-gp51 gene is crucial to the viral life cycle and infectivity, including cell entrance and the development of neutralizing antibodies,^48^^,^^49^ It has emerged as a widely used target gene for BLV diagnosis, molecular characterization, and genotyping.^12^^,^^14^^,^^16^^,^^18^^,^^19^^,^^39^^,^^50–52^ The gp51 surface glycoprotein of the *env* gene was used for the molecular detection of BLV, sequencing, and genotyping of the isolates as stated by earlier researchers around the world. The molecular prevalence of BLV was 10.88%, which differed from earlier studies carried out in Pakistan and other nations.^11^^,^^14^^,^^18^^,^^39^^,^^45^^,^^52–54^ It can be explained by variations in sample techniques and BLV detection methods. The prevalence of BLV infection reported in this study may not precisely represent the actual prevalence in the study areas, as samples were not randomly collected from individual animals but rather obtained from various cattle farms. Due to the lack of herd stratification, the different herd sizes, and the inconsistent numbers of samples taken from specific herds, the sampling system used in this study was unable to accurately represent the cattle population in the analyzed areas.^11^ The current study, however, shows that BLV infection is widespread in the Khyber Pakhtunkhwa region’s cattle.

A total of four PCR-positive samples were sequenced and subjected to multiple alignment and phylogenetic analysis using Bioedit and MEGA X, respectively. The main purpose of sequencing was to determine the genotypes of the current study BLV isolates. The sensitivity and specificity of the nested PCR assay used in this study were determined based on previously published methods and validated through positive and negative controls. The assay has been reported to have high specificity for BLV detection. However, as with any PCR-based method, there remains a possibility of false positives due to contamination or nonspecific amplification, as well as false negatives due to low viral load, PCR inhibitors in the samples, or mutations in the primer-binding regions. To minimize these risks, we included appropriate controls in each run, used DNA extraction and PCR protocols optimized for sensitivity, and ensured careful handling of samples to prevent contamination.

Recent phylogenetic analyses employing the BLV env gene sequences from isolated isolates have shown that 11 genotypes are distributed globally.^15^^,^^16^^,^^18^^,^^55^^,^^56^ The nucleotide and amino acid sequences of the gp51 gene serve as valuable genomic markers for BLV in studies on geographical distribution. Analysis of BLV gp51 env gene sequences from different regions worldwide has identified distinct genetic groups associated with specific geographic origins.^57^^,^^58^

A total of 26 sequences that were closely related to the sequences used in the current investigation were retrieved from NCBI and evaluated phylogenetically for genotyping of the BLV isolates. The sequences of the current study (OP851371-74) showed that they are clustered and close to Japanese isolates. The majority of sequences in the phylogenetic tree were typed as genotype I and only two other genotypes from Argentina (G6b) and Thailand (G10) have some relationship to the sequences used in the current investigation. Genotype I has been previously reported by Ramiz et al.^39^ and Rola-Łuszczak et al.^12^ as a prevalent genotype of BLV in Pakistan, which is in accordance with the findings of the current study. The sequences of the study are closely related to GI isolates from Japan which may be due to the reason that most cattle were imported from USA to Japan and also to Pakistan. The current study’s sequences were closely packed in the G1 genotype, and their similarity levels varied from 99 to 100 percent, which is evident from the fact that genotype G1 has one of the lowest levels of overall variability compared to other BLV genotypes.^59^ It can be hypothesized that the close phylogenetic clustering of G1 sequences in this study may indicate their origin from an early virus strain introduced to hosts in the study area. Rodriguez et al.^60^ suggested that global human and animal movement has facilitated the spread of BLV genotypes, with cattle purchases from infected herds being a significant risk factor in BLV transmission.^38^ The G1 genotype has been detected in regions such as the United States, South America, Asia, and Australia. Therefore, it is plausible that this genotype was introduced to Pakistan through the importation of exotic cattle and bull semen from BLV-endemic countries. A recent study by Moe et al.^61^ on the emergence of genotype G1 in infected cattle in Myanmar supports a similar conclusion. Genotype 1 has consistently been identified as the most prevalent worldwide, with its presence reported in over 10 countries, including Korea, Japan, the United States, Argentina, Costa Rica, Brazil, Uruguay, Australia, Iran, and Germany.^14^^,^^52^

## Conclusion

Based on the molecular epidemiology analysis of BLV, the study confirms that the virus is widespread in the study area. Phylogenetic analysis of the *gp51* gene fragment revealed that genotype G1 is the most prevalent in Khyber Pakhtunkhwa. These findings provide valuable insights into the genetic diversity of BLV in the region, contributing to a better understanding of its epidemiology and informing future diagnostic and control strategies.

## Data Availability

The data of the current experiment can be obtained from corresponding author when needed. The relevant data is provided in the paper. However, any other relevant data/information regarding the research would be provided.

## AI information

The paper has been corrected with the help of AI (chatgpt) for language correction
